# A Novel Brighter Bioluminescent Fusion Protein Based on ZZ Domain and *Amydetes vivianii* Firefly Luciferase for Immunoassays

**DOI:** 10.3389/fbioe.2021.755045

**Published:** 2021-10-18

**Authors:** Vadim R. Viviani, Jaqueline Rodrigues Silva, Paulo Lee Ho

**Affiliations:** ^1^ Department of Physics, Chemistry and Mathematics, Federal University of São Carlos (UFSCar), Sorocaba, Brazil; ^2^ Graduate Program of Biotechnology and Environmental Monitoring, Federal University of São Carlos (UFSCar), Sorocaba, Brazil; ^3^ Núcleo de Produção de Vacinas Bacterianas, Centro BioIndustrial, Instituto Butantan, São Paulo, Brazil

**Keywords:** luciferase, immunoassays, Amydetes vivianii, SARS-CoV-2, western blot, ZZ-domain

## Abstract

Immunoassays are widely used for detection of antibodies against specific antigens in diagnosis, as well as in electrophoretic techniques such as Western Blotting. They usually rely on colorimetric, fluorescent or chemiluminescent methods for detection. Whereas the chemiluminescence methods are more sensitive and widely used, they usually suffer of fast luminescence decay. Here we constructed a novel bioluminescent fusion protein based on the N-terminal ZZ portion of protein A and the brighter green-blue emitting *Amydetes vivianii* firefly luciferase. In the presence of D-luciferin/ATP assay solution, the new fusion protein, displays higher bioluminescence activity, is very thermostable and produces a sustained emission (t_1/2_ > 30 min). In dot blots, we could successfully detect rabbit IgG against firefly luciferases, Limpet Haemocyanin, and SARS-CoV-2 Nucleoprotein (1–250 ng), as well as the antigen bound antibodies using either CCD imaging, and even photography using smartphones. Using CCD imaging, we could detect up to 100 pg of SARS-CoV-2 Nucleoprotein. Using this system, we could also successfully detect firefly luciferase and SARS-CoV-2 nucleoprotein in Western Blots (5–250 ng). Comparatively, the new fusion protein displays slightly higher and more sustained luminescent signal when compared to commercial HRP-labeled secondary antibodies, constituting a novel promising alternative for Western Blotting and immunoassays.

## Introduction

Bioluminescence, the emission of visible light by living organisms has been extensively used for bioanalytical purposes during the past decades, including their use as reagents for ATP and enzymatic assays, and reporter genes for bioimaging biological and pathological processes and biosensors (Viviani and Ohmiya 2006; Roda et al., 2009). In times of pandemics, sensitive and fast detection and diagnostic methods such as immunoassays are especially demanded.

Immunoassays are widely used for detection of antibodies against specific antigens in diagnosis, as well as in electrophoretic techniques such as Western Blotting. In the past, radioactive methods involving I^125^ labelled Protein A to detect antigenic proteins have been used for Western Blots and Immunoassays (Kessler, 1975, 1981; McConahey and Dixon, 1980). Later, the radioactive methods were replaced by safer colorimetric, fluorescent or chemiluminescent methods.

The chemiluminescence methods are in general more sensitive and specific. They usually rely on the conjugation of HRP to a secondary antibody which recognizes IgG, which upon mixing with the chemiluminescent substrate solution, consisting of luminol or a derivative and H_2_O_2_, emits a blue chemiluminescence (Nesbitt and Horton, 1992).

The need of novel sensitive chemiluminescence immunoassays is especially important in times of emerging viruses and pandemics of SARS-CoV-2. As an example of the wide use of chemiluminescence immunoassays for SARS-CoV-2, a recent report shows that among the current 54 commercially available antibody-based assays for SARS-CoV-2, 13 of them are luminescent and 10 are chemiluminescent. The chemiluminescent tests display between 66 and 75% sensitivity for IgM, and 75–100% for IgG (Kojouri, 2020). Methods and reagents for automated chemiluminescence enzyme immunoassays for SARS-CoV-2 nucleocapsid protein and spike proteins are also being developed.

Bioluminescence based immunoassays consisting on the fusion of luciferases or photoproteins to antigen or an antibodies were also proposed, but in general, they were not so popular like the chemiluminescent ones. Luciferase from bacteria (*Vibrio harveyi*) was fused to protein A showing its applicability in bioluminescent immunoassays (Lindbladh et al., 1991). The photoprotein obelin from *Obelia longissima* (Cnidaria) was attached to ZZ-domain of protein A demonstrating the possibility of its application in immunoassays (Frank et al., 1995). Obelin was also conjugated to anti-thyroid hormones (human thyrotropin and thyroxine), and the sensitivity of these bioluminescent immunoassays were similar to those using radioisotopes (Frank et al., 2004). Kobatake et al. (1993) produced a fusion protein based on the protein A fused to N-terminal deleted *Photinus pyralis* firefly luciferase. However, despite being antigenically active, this construct displayed weaker luminescent activity than wild-type firefly luciferase. Later, the authors fused protein A to the full-length firefly luciferase, obtaining a more active construct, with high affinity for IgG, detecting up to 5 pg of tumor marker a-fetoprotein (AFP) (Zhang et al., 2000).

More recently, the nanoluciferase from *Oplophorus gracilirostris* (deep-sea shrimp) was fused to a nanobody against aflatoxin B_1_, a potentially carcinogenic mycotoxin produced by fungi in cereals, resulting in an attractive, simple, and rapid analytical tool for quantification of the pollutants in commercial foods (Ren et al., 2019). An immunoassay for antibodies against SARS-CoV-2 proteins based on the fusion of viral S e N protein fragments with NanoLuc luciferase was also developed. This method was specific to quantify the levels of SARS-CoV-2 antibodies in patients (Haljasmägi et al., 2020).

A quantitative bioluminescence immunoassay for immunohistochemistry based on *Cypridina* luciferase conjugated secondary antibody and its luciferin, was also developed and successfully used to detect the tumor marker carcinoembryonic antigen (Wang et al., 2020). Bioluminescent sensors to detect multiple antibodies based on microfluidics and BRET were also proposed (Kosuke et al., 2020).

Although nowadays most immunoassays use IgG based secondary antibodies, protein A still remains a useful and cheaper alternative, especially for affinity purification of antibodies (Huang et al., 2006). Protein A was first isolated from *Staphylococcus aureus*, and due to its high affinity for the portion Fc of immunoglobulins, has been extensively used in immunoassays. Furthermore, the small ZZ portion of protein A, can be used to construct smaller fusion proteins which are highly expressed in bacteria (Drevet et al., 1997).

In order to develop efficient bioluminescence based immunoassays, here we report the construction and uses of a novel bioluminescent fusion protein based on the ZZ portion of protein A and a brighter luciferase arising from *Amydetes vivianii* firefly, which emits a more blue-shifted emission than other firefly luciferases (Viviani et al., 2011; Pelentir et al., 2019). The new fusion protein can be successfully used for CCD imaging and photographical detection of several primary antibodies in immunoassays and Western Blots, including the detection of Anti-SARS-CoV-2 nucleoprotein.

## Material and Methods

**Reagents.** SARS-CoV-2 Nucleoprotein and Anti-SARS-CoV-2-Nucleoprotein were obtained from CusaBio (Houston, United States); Anti-goat firefly luciferase, firefly D-luciferin potassium salt was obtained from Promega (Madison, United States); Anti-rabbit Hemocyanin and CoA were obtained from Sigma (St Louis, United States). Western Blotting chemiluminescent detection kit was obtained from GE Healthcare (Chicago, United States).

**cDNAs and constructions.** The chimeric protein DNA was constructed by ligating protein A ZZ fragment with the N-terminal of *Amydetes vivianii* firefly luciferase cDNA inside the pCold vector (Takara, Japan). For this purpose, ZZ DNA fragment was amplified from the plasmid pCP (Drevet et al., 1997), using primers containing *Nde*I restriction sites (ZZ-For) GAT ATA CAT ATG GCG CAA CAC and (ZZ-Rev) GCC GCA TAT GGA TCC ATG GAC TAG TGA TC. The amplification product was digested with *Nde*I, purified, and then ligated to the *Nde* I-digested and dephosporylated pCold vector containing the *Amydetes* luciferase cDNA (pC-Amy), using Takara Ligation kit. The ligation was used to transform *E. coli* XL1Blue, the recombinant colonies were induced with IPTG O/N, sprayed with D-luciferin and screened by photodetection using CCD camera (ATTO, Japan). The construction was confirmed by digestion with *Nde I*, by PCR using ZZ and pCold-F primers, and finally by DNA sequencing.

**Protein expression and purification.** The vectors containing the wild-type *Amydetes vivianii* (pC-AmyLuc) and chimeric protein (pC-ZZ-AmyLuc) were used to transform *E. coli* BL21 cells. For ZZ-AmyLuc and wild-type luciferase expression, transformed *E. coli* BL21 (DE3) cells were grown in 100–1,000 ml of LB medium at 37°C up to OD_600_ = 0.4, and then induced at 18°C with 0.4 mM IPTG during 18 h. Cells were harvested by centrifugation at 2,500 g for 15 min and resuspended in extraction buffer consisting of 0.10 M sodium phosphate buffer, 1 mM EDTA, and 1% Triton X-100, 10% glycerol and protease inhibitor cocktail (Roche, Switzerland), pH 8.0, lysed by ultrasonication and centrifuged at 15,000 g for 15 min at 4°C. The N-terminal histidine-tagged fusion protein and wild-type *Amydetes* firefly luciferase were purified by agarose-Nickel affinity chromatography (Wash buffer: 50 mM Phosphate pH 7.0; 300 mM NaCl, 20 mM imidazole and Elution buffer 50 mM Phosphate pH 7.0; 300 mM NaCl, 250 mM imidazole) followed by dialysis (25 mM TRIS-HCl pH 8.0, 10 mM NaCl, 1 mM EDTA, 2 mM DTT, and 15% glycerol), as described (Pelentir et al., 2019). The concentrations of purified luciferases were between 0.5 and 1 mg/ml, and the estimated purity, according to SDS-PAGE gels were about 90%.

**Luminometric assays for bioluminescence and chemiluminescence.** Luciferase bioluminescence and HRP chemiluminescence activities were measured using an AB2200 (ATTO; Tokyo, Japan) luminometer. The assays for luciferase activity were performed by mixing 5 µl of 40 mM ATP/80 mM MgSO_4_ solution with a solution consisting of 5 µl of luciferase or chimeric protein and 90 µl of 10 mM luciferin in 0.10 M Tris-HCl pH 8.0 at 22 C. For commercial rabbit horseradish peroxidase (HRP) conjugated antibody chemiluminescence activity, 5 µl of diluted HRP-conjugated antibody were mixed with 50 µl of substrate solution 1 and 50 µl of substrate solution 2. All measurements were done in triplicate for at least three independent luciferase preparations, and averages and the standard deviations were reported in Table 1.

**TABLE 1:** Luminescence properties of ZZ-AmyLuc fusion protein, wild-type *Amydetes vivianii* luciferase and HRP-conjugated secondary antibody from GE Healthcare.

luciferase	Specific Activity (10^12^cps/mg)	Half-life at 37°C (h)	*k*_cat_ (10^−6^cps)	t_1/2_ (min)	λmax (nm)
*Amydetes vivianii* luciferase	6.0	12	109	12 ± 0.325	550
ZZ-AmyLuc	5.1 ± 0.8	18	150	18.7 ± 2.5/28 ± 2.8^a^	550
HRP- secondary antibody-	2.5 ± 0.54	—	—	10.3 ± 0.76	460

a Half-life of the luminescence kinetics obtained in the presence of CoA.

**Kinetics**. The kinetics of luminescence reactions were measured using a TD-III luminometer (Japan). The half-life of luminescence (t_1/2_) was the time necessary to reach half intensity from the peak of intensity, and was calculated form the average of three assays. In the standard assay, 5 µl of 80 mM MgSO4 and 40 mM ATP solution were mixed to a solution consisting of 5 µl of luciferase and 90 µl of 10 mM D-luciferin in 0.10 M Tris-HCl buffer pH 8.0.

**CCD Imaging.** Dot blot and Western blotting chemiluminescence detection was done using a LightCapture II CCD camera (ATTO, Japan) at different exposure times.

**Smartphone and photographic imaging.** For photographical imaging of immunoassays, we used a Canon Ti5, and for smartphone imaging we used a dark-adapted box for smartphones cameras, and a Samsung Galaxy S10 *Plus* cell phone.

**Immuno-dot.** For dot blotting using distinct antibodies, 1 µl of the antibodies at concentrations ranging from 1 to 500 ng/μl were spotted over nitrocellulose (NC) membrane. Then the membrane was incubated with blocking solution (5% dry milk in PBS-Tween) for 1 h, followed by 2 fast washes and 1 wash for 10 min in PBS-Tween (0.5 mM Na_2_HPO_4_; 0.5 mM NaH_2_PO_4_; 10 mM NaCl; 0.1% Tween pH 7.4), incubation with the ZZ-AmyLuc (1/400–1/1,000 dilutions) or rabbit HRP-labelled secondary antibody (1/1,000) during 1 h, and then washed again. Finally, 0.5–1 ml of luciferase assay solution (0.5 mM D-luciferin, 2 mM ATP, 4 mM MgSO_4_, 5 mM DTT, 0.25 mM CoA in 0.10 M Tris-HCl buffer pH 8.0) or HRP substrate solution mix, were pipetted over the NC membrane, the membrane was left standing during 1 min, and finally exposed to CCD camera LightCapture II (ATTO, Japan) or photographic detection.

**Western Blotting.** For Western Blotting, SDS-PAGE electrophoresis of different protein samples was performed, and then the proteins were electro-transferred from the SDS-PAGE gel to NC membranes. The NC membranes were then threated as described for Dot Blotting, first by incubation with blocking solution, followed by incubation with the primary antibodies, secondary HRP-labeled antibody or ZZ-AmyLuc fusion protein, and finally the luminescence revealed upon addition of luciferase assay solution for ZZ-AmyLuc chimeric protein, or substrate solution 1 and 2 mix for commercial HRP-labelled secondary antibody.

## Results and Discussion

### ZZ-AmyLuc Molecular Properties

**FIGURE 1:**
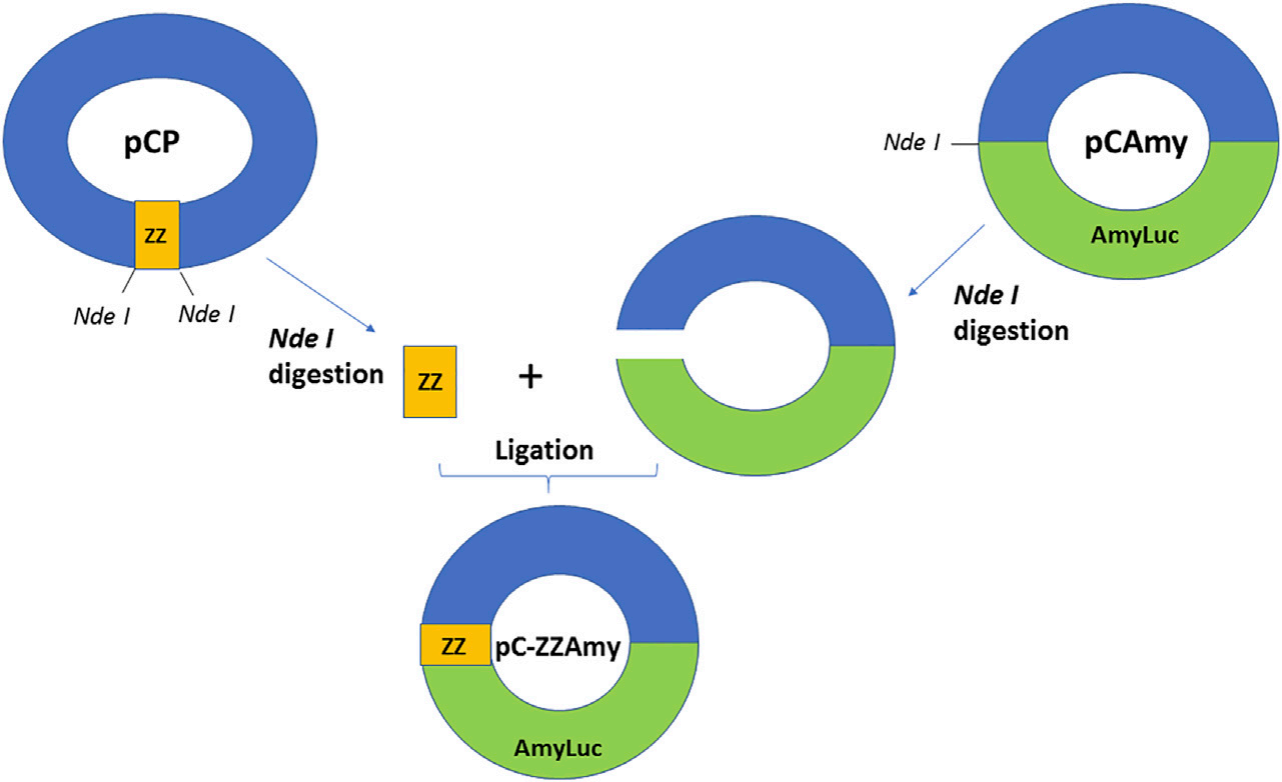
Chimeric ZZ-Amy protein DNA construction scheme.

We constructed a fusion protein using the cDNA of the ZZ portion of protein A at the N-terminus of a brighter luciferase (Figure 1), originally cloned from *Amydetes vivianii* firefly from campus of Sorocaba of Federal Univ. of São Carlos, which emits one of the most blue-shifted emissions among firefly luciferases (Viviani et al., 2011). The chimeric protein ZZ-AmyLuc DNA consists of 423 bp of the ZZ portion of protein A and the full-length cDNA of *Amydetes vivianii* luciferase inside the pCold vector (pC-ZZ-AmyLuc). The coded chimeric protein has therefore 699 amino-acid residues, consisting of a 11 amino-acid residues N-terminal fragment containing a 6 His-tag conferred by the pCold vector, followed by the 141 amino-acid residues of the ZZ portion of protein A, and finally the 547 residues of *Amydetes vivianii* firefly luciferase. The protein has a calculated MW of 77.879 Da and pI = 6.24.

### Expression and Purification

**FIGURE 2:**
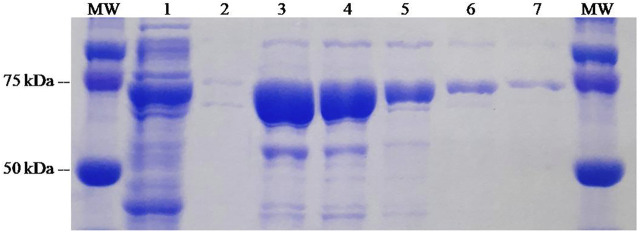
SDS-PAGE of the purification process of ZZ-Amy fusion-protein: (MW) molecular weight standard; (1) crude extract of *E. coli* expressing ZZ-Amy; (2) void volume; (3) elution 1; (4) elution 2; (5) elution 3; (6) elution 4; (7) elution 5.

The ZZ-AmyLuc fusion protein, as well as the wild-type luciferase, were expressed in bacteria and purified by nickel-agarose affinity chromatography according to published procedures (Pelentir et al., 2019). In the SDS-PAGE, the polypeptide displayed a MW of ∼75 kDa, and the final purification yield was ∼10 mg/L of bacterial culture (Figure 2).

### Bioluminescent Properties of the Fusion Protein

Table 1 summarizes the bioluminescence properties of ZZ-AmyLuc fusion protein, and compares its luminescence properties with those of wild-type luciferase and commercial GE-Helathcare chemiluminescent kit using HRP/Luminol system. The specific luminescent activity of ZZ-AmyLuc (5.10^12^ cps/mg) was similar to that of the wild-type *Amydetes vivianii* firefly luciferase (6. 10^12^ cps/mg). The catalytic constant was slightly higher for the fusion protein in relation to the wild-type luciferase.

**FIGURE 3:**
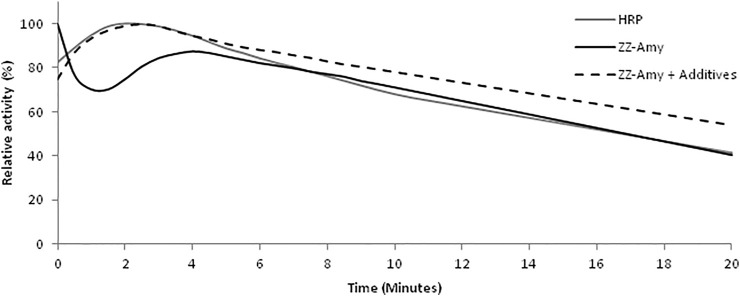
Comparison of the luminescence kinetics of the commercial chemiluminescent HRP/luminol assay and with the bioluminescent fusion protein ZZ-AmyLuc assay. The HRP/luminol chemiluminescent assay was performed by mixing 5 µl of HRP-conjugated secondary antibody with 50 µl of Solution 1 and 50 µl of Solution 2. The standard bioluminescent assay with ZZ-AmyLuc was performed by mixing 5 µl of fusion protein with 95 µl of a solution consisting of 0.5 mM D-luciferin, 2 mM ATP and 4 mM MgSO4 in 0.1 M Tris-HCl pH 8,0. The assay with additives was performed in the additional presence of 0.25 mM CoA and 5 mM DTT.

The luminescence reaction kinetics in the presence of luciferin, ATP and magnesium at pH 8.0 displays a flash with fast decay, followed by a slight increase and a very slow decay which is sustained for several minutes (Figure 3), with a half-life of ∼20 min. In the presence of CoA and DTT, however, the kinetics became slower, lasting hours, with a half-life of ∼30 min at room temperature. CoA has been shown to be a substrate of firefly luciferase, reacting by thioesterification with the potent inhibitor dehydroluciferyl-adenylate, which is responsible for the fast inhibition of the luminescence reaction and flash-like luminescence kinetics (Fraga et al., 2005). Addition of CoA causes removal of this inhibitor from the active site, resulting in a more sustained luminescence. This sustained luminescence kinetics displayed by the fusion protein allows detecting even weaker signals upon integration for longer times.

The fusion protein was also quite stable, keeping 38% of activity when incubated at 37°C during 24 h, 60% activity when incubated at 4°C during 37 days, and 84% activity when incubated at 4°C during 65 days in the presence of glycerol 15%.

Finally, the bioluminescence spectrum overlapped with that of the wild-type *Amydetes vivianii* luciferase, with an emission peak at 550 nm, and also displaying pH-sensitivity (results not shown).

## IgG Binding Properties

**FIGURE 4:**
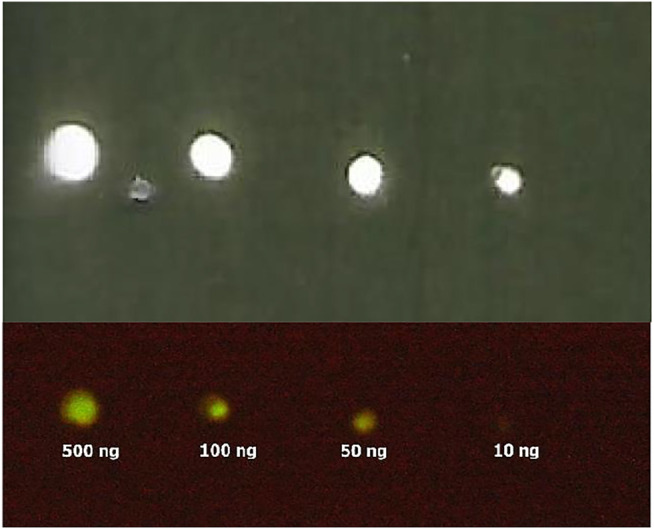
Bioluminescence immunodot showing different amounts of immobilized Anti-SARS-CoV-2 nucleoprotein using ZZ-AmyLuc fusion protein: (upper image) CCD imaging, and (lower image) photographic detection. Using CCD imaging, it is possible to detect up to 1 ng of primary antibody, whereas using photographic detection it is possible to detect up to 10 ng.

**FIGURE 5:**
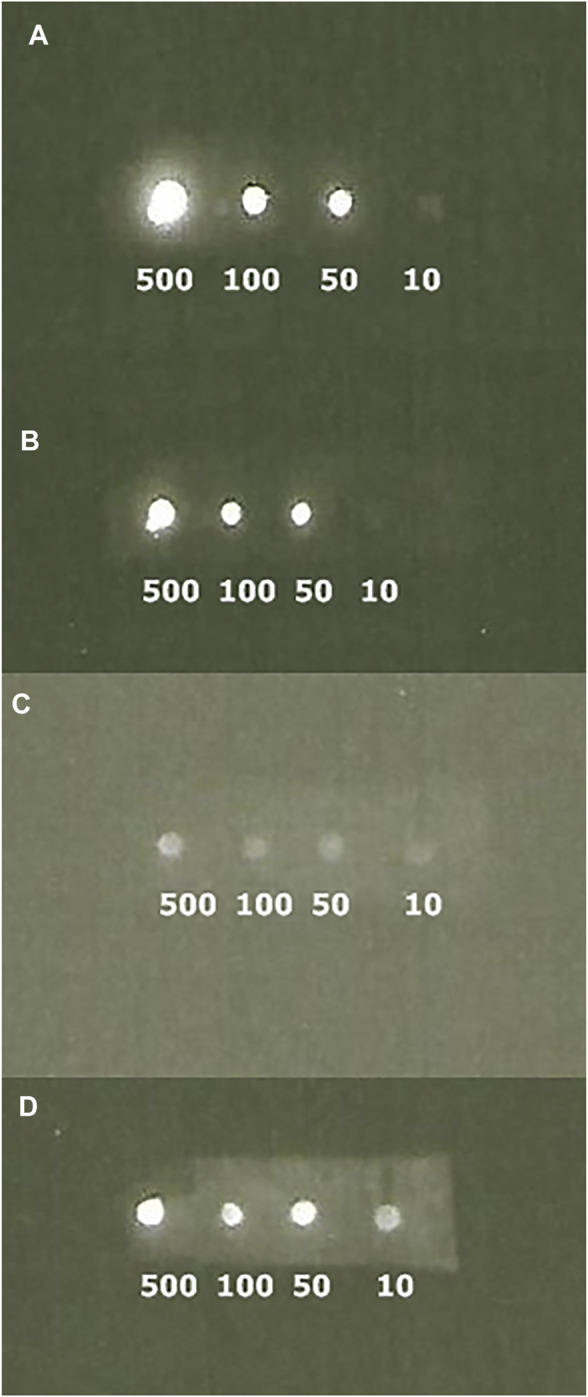
**CCD imaging of** Dot blots comparing the luminescent signal of primary polyclonal antibodies raised against limpet hemocyanin revealed with ZZ-AmyLuc fusion protein (A and B) and commercial HRP-labeled secondary antibody (C and D): **(A)** revealed with ZZ-AmyLuc fusion protein and its assay solution after 1 min exposure; **(B)** same experiment of A after 2 h incubation at room temperature and 1 min exposure; **(C)** revealed with commercial HRP-labeled secondary antibody and its assay solution from GE HealthCare after 1 min exposure and **(D)** same experiment of C after 5 min exposure.

**IgG binding activity of ZZ-AmyLuc.** The binding activity of ZZ-AmyLuc fusion protein was first analyzed by spotting primary antibodies on nitrocellulose membranes, followed by incubation with ZZ-AmyLuc or with HRP-labelled secondary antibody, and revealed by bio- and chemiluminescence using the respective assay solutions. Distinct IgGs, especially those raised from rabbits, including Anti-SARS-CoV-2 nucleoprotein and anti-hemocyanin were detected by CCD imaging (Figure 4 and Figure 5). The luminescent signal was easily detected by conventional CCD camera system (LightCapture 2 ATTO, Japan) after 1 min exposure, and was also intense enough to be photographically detected (Figure 4). Considering Figure 4, less than 1 ng of rabbit antibodies against SARS-CoV-2 nucleoprotein could be detected using CCD camera, and less than 10 ng by photographic detection.

**FIGURE 6:**
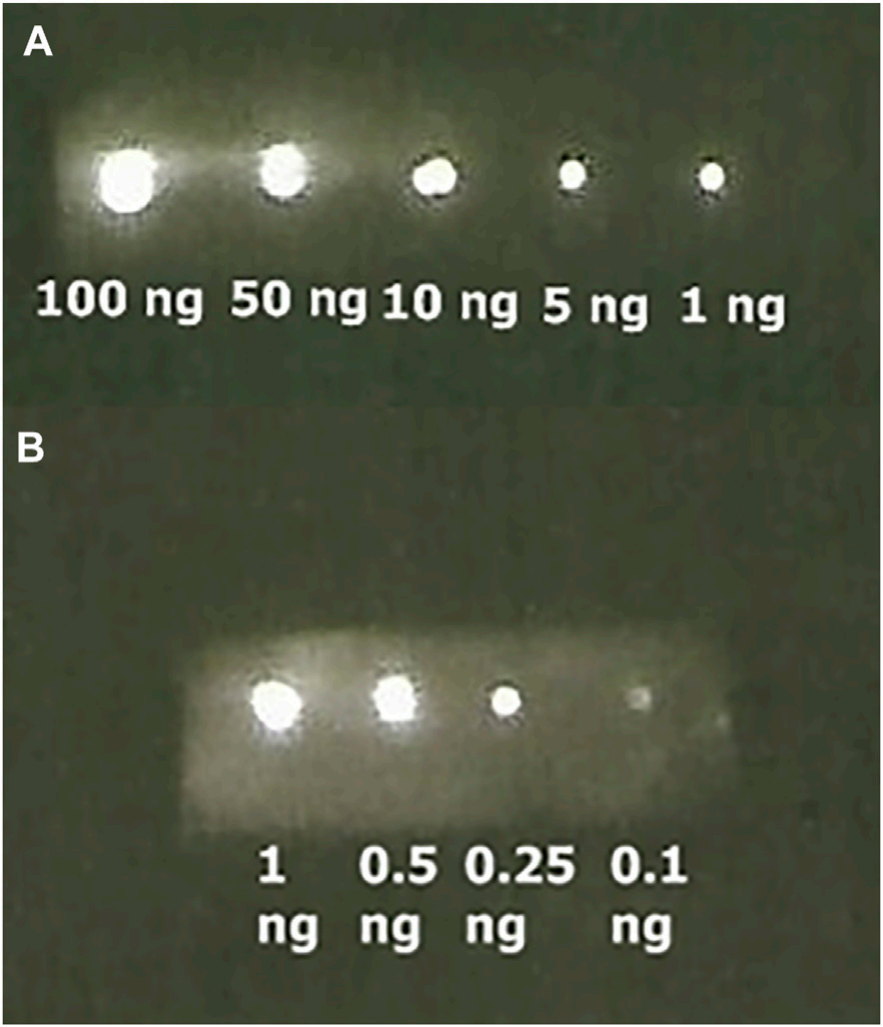
Bioluminescence immunoassay of SARS-CoV-2 Nucleoprotein bound to its respective antibody and revealed with ZZ-AmyLuc fusion protein: **(A)** from 100 ng to 1 ng exposed during 1 min, and **(B)** from 1 to 0.10 ng exposed 5 min.

Then, we performed immunoblotting by spotting SARS-CoV-2 nucleoprotein over the NC membrane, followed by incubation with its primary antibody, the ZZ-AmyLuc protein and revealed with its assay solution. Using this procedure, we could detect up to 100 pg of antigen by conventional CCD imaging (Figure 6).

**FIGURE 7:**
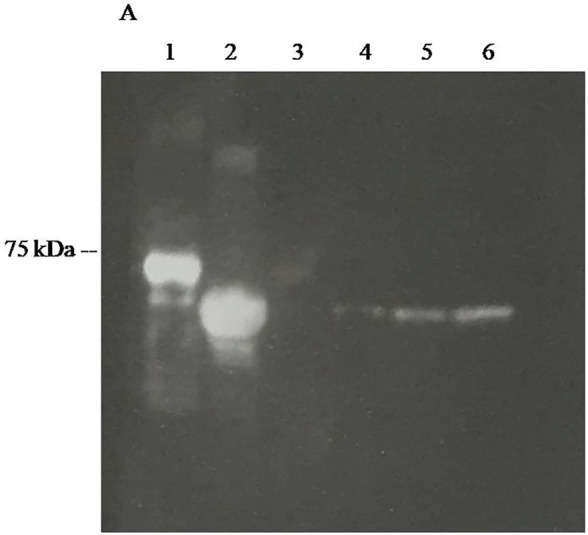
CCD imaging of Western blots firefly luciferases using polyclonal antibodies raised against *Photinus pyralis* firefly luciferase revealed using ZZ-AmyLuc fusion protein as a secondary antibody and its assay solution: (1) ZZ-AmyLuc; (2) purified WT *Amydetes vivianii* firefly luciferase; (3) 10 ng of purified *Amydetes vivianii* luciferase*;* (4) 50 ng of purified *Amydetes vivianii* luciferase*;* (5) 100 of purified *Amydetes vivianii* luciferase*;* (6) 200 ng of purified *Amydetes vivianii* luciferase.

**FIGURE 8:**
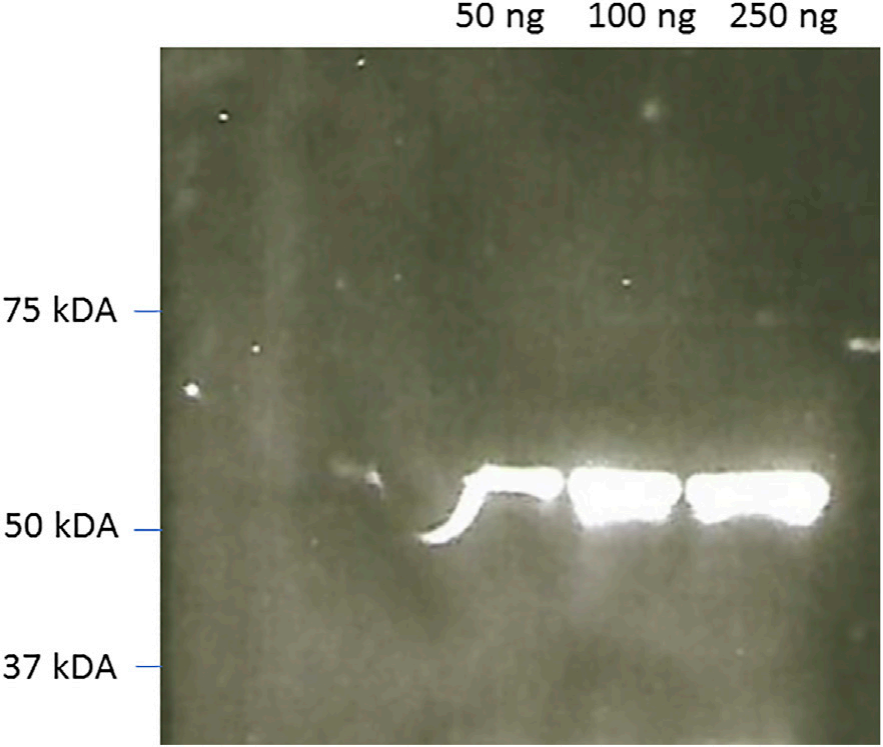
CCD imaging of Western blots of commercial SARS-Covid-2 nucleoproteín using rabbit Anti-SARS-covid and reveled using ZZ-AmyLuc fusion protein as a secondary antibody and its assay solution. Exposure time: 1 min.

**Western Blots.** In Western Blots, the fusion protein could also be effectively used to reveal commercial and home expressed firefly luciferases (Figure 7) and SARS-CoV-2 Nucleoprotein (Figure 8). The luminescent signals were intense and comparable with Western blots revealed using commercial HRP-labeled secondary antibodies.

**Comparison of ZZ-AmyLuc bioluminescent assay with commercial HRP/luminol.** As shown above, the bioluminescent immunoassays using ZZ-AmyLuc fusion protein and its assay solution with luciferin and MgATP, were brighter than the commercial chemiluminescent assay of GE HealthCare based on the HRP conjugated secondary antibody and luminol.

Whereas the relative *in vitro* luminescent activity of the HRP-based chemiluminescent assay of GE Healthcare was higher than that of the fusion protein ZZ-AmyLuc, when using the same volumes of enzyme and respective assays solutions (5 µl enzyme and 95 µl of respective assay solution), the specific activity of the ZZ-AmyLuc fusion protein was higher than that of the commercial HRP conjugated secondary antibody (Table 1). Furthermore, considering the higher sensitivity of the luminometer photomultiplier in the blue region, where the HRP/luminol reaction emits (460 nm), in relation to ZZ-AmyLuc/luciferin system which emits in the green region (550 nm), it is expected that ZZ-AmyLuc may display even higher luminescence activity than the HRP-luminol system.

The duration of the luminescent signal of ZZ-Amy fusion protein bioluminescent assay, as measured from the half-life from the peak of luminescence activity, was longer than the signal of the HRP/luminol chemiluminescent assay, with a half-life of ∼20 min in the classic assay with luciferin and ATP, and ∼30 min for the assay solution with additives, whereas the commercial HRP/luminol-based assay had a half-life of only ∼10 min. These properties indicate that the luminescence signal of Western Blots and immunoassays could be detected later than 30 min after the addition of the reagent, whereas HRP assay can be used not much longer than 10 min. In practice, the intensity of the luminescence signal emitted by the immobilized complex of ZZ-AmyLuc-primary antibody-antigen complex in dot blots, was still very high after 2 h from the beginning of the assay. Finally, the reagent Amy-Luc also showed very good stability in buffered solution at 4°C, keeping the full activity for more than 2 months.

We could not directly compare the bioluminescence activity and properties of this ZZ-AmyLuc fusion protein with those of the construct of *Photinus pyralis* firefly luciferase/protein-A described by Kobatake et al. (1993). However, considering that: 1) the reported bioluminescence activity of *P. pyralis* firefly luciferase construct is nearly the same of the respective wild-type luciferase; 2) that *A. vivianii* wild-type luciferase has similar catalytic constant than *P. pyralis* luciferase (Pelentir eta l., 2020), and 3) that the construct ZZ-AmyLuc described here has slightly higher catalytic constant than the respective wild-type luciferase, we assume that ZZ-Amy-Luc also has higher bioluminescence activity than *P. pyralis* firefly luciferase-Protein A construct.

Altogether, these properties make the ZZ-AmyLuc fusion protein an attractive brighter and a stable competitive luminescent reagent for immunoassays. One drawback of this system is the differential recognition of immunoglobulins by protein A. It is known that protein A may usually binds effectively to distinct IgGs from distinct biological sources (rabbit, goat, mouse, human), but not to IgM, restricting its applicability to IgGs. However, the construction and properties of the fusion-protein reported here, besides being useful to detect IgGs, demonstrates the potential of *Amydetes* luciferase for construction of novel bioluminescent fusion protein with high affinity for antibodies. Another issue to be considered for the potential commercial use of this fusion protein in immunoassays, is the elevated cost of the substrate D-luciferin and assay solutions. However, although the luminol and H_2_O_2_ solutions are cheaper than D-luciferin, an estimate made by comparing the number of assays performable with GE-Healthcare Western Blotting kit and with the D-luciferin assay solution prepared here, in the absence of the very expensive CoA, showed that the price for assay is cheaper for the luciferin solution than that of luminol solution 1 and 2.

**FIGURE 9:**
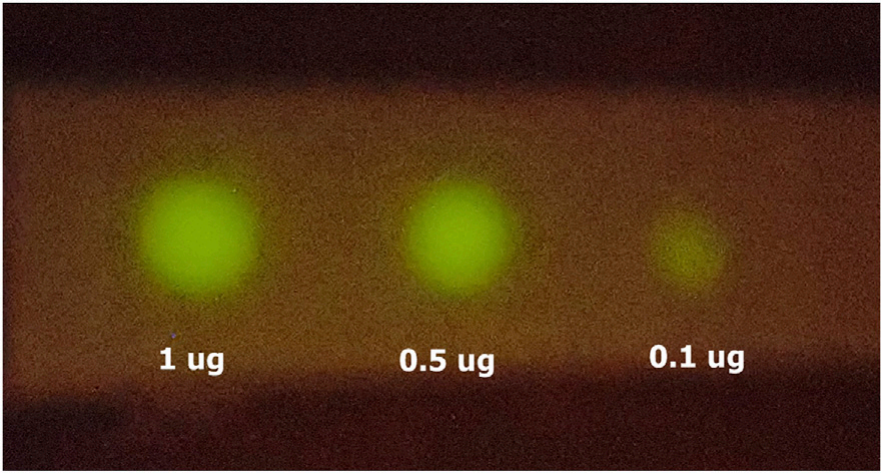
Smartphone imaging of spotted antibodies against SARS-CoV-2 nucleoprotein, revealed with ZZ-AmyLuc and its assay solution. The image was obtained with a Galaxy S10 Plus smartphone.

**Smartphone detection**. The use of smartphones for clinical diagnosis using bioluminescence is an increasing trend for point-of-care medicine and biosensors (Roda et al., 2014; Tomimuro et al., 2020). We have imaged the bioluminescent signal emitted by dot blots with spotted SARS-CoV-2 nucleoprotein and haemocyanin primary antibodies, and revealed with ZZ-AmyLuc fusion protein and its assay solution, using a Galaxy S-10 *plus* cell phone CCD camera. Using this procedure, we could detect up to 100 ng of antibodies (Figure 9). Considering that the CCD camera of this smartphone displays an average sensitivity to light among the smartphones CCD cameras found in the market, the experiment shown here also attests the feasibility of using smartphone technology for bioluminescent immunoassays using ZZ-AmyLuc fusion protein.

## Concluding Remarks

We constructed a novel bioluminescent fusion protein based on ZZ portion of protein A and the brighter *Amydetes vivianii* firefly luciferase for immunoassays and Western Blots. The new fusion protein displayed superior luminescence properties, effectively recognizing rabbit primary antibodies in immunoassays and Western Blots. The fusion protein could be successfully used to detect less than 1 ng of primary antibody against SARS-CoV-2 nucleoprotein by CCD imaging, 10 ng by photography, and up to 100 pg of immobilized SARS-CoV-2 nucleoprotein using its respective antibody. The luminescence signal emitted by the fusion protein in dot blots is very bright and lasts enough time to be detected until to 2 h after the beginning of the assay. The methodology is also sensitive enough to allow detection of up to 100 ng of antibodies using an average smartphone camera. Altogether, these properties make ZZ-AmyLuc a novel promising bioluminescent secondary antibody for immunoassays and Western Blots.

## Data Availability

The original contributions presented in the study are included in the article/supplementary material, further inquiries can be directed to the corresponding author.
